# Red Cell Distribution Width Association with Subclinical Cardiovascular Disease in Patients with Rheumatoid Arthritis

**DOI:** 10.3390/jcm12206497

**Published:** 2023-10-12

**Authors:** Marta González-Sierra, Alejandro Romo-Cordero, Juan C. Quevedo-Abeledo, Adrián Quevedo-Rodríguez, Fuensanta Gómez-Bernal, Antonia de Vera-González, Raquel López-Mejías, Candelaria Martín-González, Miguel Ángel González-Gay, Iván Ferraz-Amaro

**Affiliations:** 1 Division of Hospitalization-at-Home, Hospital Universitario de Canarias, 38320 Tenerife, Spain; martagses@gmail.com; 2 Division of Internal Medicine, Hospital Universitario de Canarias, 38320 Tenerife, Spain; alexromo96co@gmail.com (A.R.-C.); mmartgon@ull.edu.es (C.M.-G.); 3 Division of Rheumatology, Hospital Universitario Dr. Negrín, 35010 Las Palmas de Gran Canaria, Spain; quevedojcarlos@yahoo.es (J.C.Q.-A.); adrian-ce@hotmail.es (A.Q.-R.); 4 Division of Central Laboratory, Hospital Universitario de Canarias, 38320 Tenerife, Spain; fuensanta95@gmail.com (F.G.-B.); adeverag@gmail.com (A.d.V.-G.); 5 Epidemiology, Genetics and Atherosclerosis Research Group on Systemic Inflammatory Diseases, Hospital Universitario Marqués de Valdecilla, Instituto de Investigación Sanitaria Marqués de Valdecilla (IDIVAL), 39011 Santander, Spain; rlopezmejias78@gmail.com; 6 Internal Medicine Department, Universidad de La Laguna, 38200 Tenerife, Spain; 7 Department of Medicine and Psychiatry, Universidad de Cantabria, 39005 Santander, Spain; 8 Division of Rheumatology, IIS-Fundación Jiménez Díaz, 28040 Madrid, Spain; 9 Division of Rheumatology, Hospital Universitario de Canarias, 38320 Tenerife, Spain

**Keywords:** rheumatoid arthritis, red cell distribution width, cardiovascular disease, SCORE2

## Abstract

Red cell distribution width (RDW) is a measure of the variation in mean corpuscular volume that reflects the degree of anisocytosis on the peripheral blood smear. RDW value variation has been implicated in several disorders including chronic inflammatory processes and cardiovascular (CV) diseases. In the present work, our objective was to study the relationship that RDW has with the characteristics of the disease in patients with rheumatoid arthritis (RA), focusing on CV risk factors and subclinical atherosclerosis. A cross-sectional study was conducted that included 430 patients with RA and 208 controls matched by sex and age. Complete blood count, including RDW, was assessed. Multivariable analysis was performed to analyze the relationship of RDW with RA disease characteristics, subclinical carotid atherosclerosis, and traditional CV factors, including a comprehensive profile of lipid molecules and insulin resistance and beta cell function indices. After multivariable adjustment, the RDW was significantly higher in RA patients compared with controls (beta coefficient 1.0 [95% confidence interval 0.2 to 1.8] %, *p* = 0.020). Furthermore, although the erythrocyte sedimentation rate showed a positive and significant relationship with RDW, this association was not found with C-reactive protein and interleukin-6. A positive and independent relationship was observed between DAS28-ESR disease activity score and RDW. However, no association was found between the RDW and other disease activity scores that do not include erythrocyte sedimentation rate in their formula. The SCORE2 CV risk algorithm was positively and significantly associated with higher RDW values. Likewise, a negative relationship was found between RDW with total cholesterol and low-density lipoprotein cholesterol, and a positive relationship was found between RDW and insulin resistance indices. In conclusion, RDW values are higher in RA patients compared to matched controls. Although the relationship of RDW with disease activity was not consistent, RDW shows associations with subclinical CV disease risk factors, including dyslipidemia and insulin resistance, and with the SCORE2 CV disease-risk prediction algorithm.

## 1. Introduction

Red blood cell distribution width (RDW) is a measure of the variation in mean corpuscular volume, which is reflected by the degree of anisocytosis in the peripheral blood smear. RDW is calculated as the coefficient of variation or the standard deviation of the mean corpuscular volume distribution curve [1]. The reference range typically spans between 12 and 15% [2]. A high RDW implies a large variation in red blood cell sizes. Therefore, an elevated RDW can be seen in iron deficiency anemia, transfused anemia, myelodysplastic syndromes, and hemoglobinopathies, whereas a normal to slightly elevated RDW can be seen in the thalassemia trait and anemia of chronic disease/anemia of inflammation. Abnormalities in the RDW curve may indicate certain types of anemia. In this sense, an extension of the left shoulder to the curve (red blood cell population with smaller volumes) may indicate microspherocytes or schistocytes. In contrast, a shoulder on the right side usually corresponds to an extremely large population of red blood cells or reticulocytes and may indicate an agglutination of red blood cells [3].

Newly available evidence indicates that RDW disruption occurs in various human disorders like cardiovascular (CV) disease, venous thromboembolism, cancer, diabetes, community-acquired pneumonia, chronic obstructive pulmonary disease, liver and kidney failure, and other acute or chronic conditions [4]. Of greater significance, RDW is currently being recognized as a potent and autonomous predictor of mortality within the general population [5,6,7,8]. However, it remains uncertain whether an elevated RDW value serves as a genuine risk factor or simply represents a side effect of an underlying biological and metabolic disparity. No definitive conclusions have currently been reached in this regard.

Rheumatoid arthritis (RA) is a systemic inflammatory disease characterized by peripheral polyarthritis. Common hematological complications associated with RA include anemia, thrombocytosis, and cytopenias [9]. Besides joint manifestations, patients with RA often experience involvement in other organs such as the eye, lungs, and heart. However, the link between RDW and disease characteristics in RA remains a topic of debate, as most studies are based on small cohorts of patients and without performing multivariable analyses. To address this issue, we have conducted a study based on a substantial group of RA patients. In this regard, our study encompasses information on indicators of disease activity, inflammation status, and concurrent conditions, including lipid profiles, insulin resistance, subclinical atherosclerosis, and cardiovascular risk based on the Systematic Coronary Risk Assessment (SCORE)2 algorithm. Subsequently, we have analyzed the relationship between all these characteristics and the RDW.

## 2. Materials and Methods

### 2.1. Study Participants

This was a cross-sectional study that included 430 consecutively recruited RA patients and 208 sex- and age-matched controls. All patients with RA were 18 years or older and met the 2010 ACR/EULAR classification criteria [10]. They had been diagnosed by rheumatologists and were periodically followed up at rheumatology outpatient clinics. For inclusion in the present study, the duration of RA disease was required to be ≥1 year. Since glucocorticoids are often used in the treatment of RA, patients taking prednisone or an equivalent dose of ≤10 mg/day were allowed to participate. Controls were community-based and recruited by general practitioners in primary care centers. However, controls with a history of any inflammatory rheumatic disease were excluded. None of the controls were receiving glucocorticoids. Patients and controls were excluded if they had a history of CV events like myocardial infarction, angina, and stroke, a glomerular filtration rate <60 mL/min/1.73 m^2^, a history of cancer or any other chronic disease such as hypothyroidism, heart or respiratory diseases, nephrotic syndrome, as well as evidence of active infection. None of the patients and controls had a hematological disease such as aplasia or myeloproliferative disorders. The study protocol was approved by the Institutional Review Committee at Hospital Universitario de Canarias and at Hospital Universitario Doctor Negrín (both in Spain), and all subjects provided informed written consent (approval no. 2019-452-1). All research was performed in accordance with relevant guidelines/regulations and in accordance with the Declaration of Helsinki

### 2.2. Data Collection and Laboratory Assessments

Individuals included in the study completed a questionnaire on CV risk factors and medication use and underwent a physical examination. Body mass index (BMI) (the weight in kilograms divided by the square of the height in meters), abdominal circumference, and systolic and diastolic blood pressure were assessed under standardized conditions. Information regarding smoking status, diabetes, and hypertension was obtained from the questionnaire. Medical records were reviewed to ascertain specific diagnoses and medications. The Sysmex-XN automated blood cell analyzer (Sysmex, Kobe, Japan) was used to measure blood cell counts. The Sysmex instrument uses the standard deviation, and not the coefficient of variation, of the MCV distribution curve for the calculation of RDW. Cholesterol, triglycerides, and HDL-cholesterol were measured using an enzymatic colorimetric assay. LDL-cholesterol was calculated using the Friedewald formula. Dyslipidemia was defined if one of the following was present: total cholesterol > 200 mg/dL, triglycerides > 150 mg/dL, HDL cholesterol < 40 in men or <50 mg/dL in women, or LDL cholesterol > 130 mg/dL. A standard technique was used to measure the erythrocyte sedimentation rate (ESR) and high-sensitivity C-reactive protein (CRP). Human interleukin 6 (IL-6) was measured using the electrochemiluminescence immunoassay method (Roche Diagnostics, Indianapolis, IN, USA). Serum apolipoprotein C-III was assessed using a sensitive sandwich enzyme-linked immunosorbent assay (Elabscience, Houston, TX, USA). The homeostatic model assessment (HOMA) method was performed to determine IR. Briefly, the HOMA model enabled an estimate of insulin sensitivity (%S) and β-cell function (%B) from fasting plasma insulin, C peptide, and glucose concentrations. In this study, we used HOMA2, the updated-computer HOMA model [11]. Disease activity in patients with RA was measured using the Disease Activity Score (DAS28) in 28 joints [12], the Clinical Disease Activity Index (CDAI) [13], and the Simple Disease Activity Index (SDAI) [14]. DAS28-ESR and DAS28-CRP were categorized according to clinical remission (<2.6), low (>2.6 to 3.2), moderate (>3.2 to 5.1), or high disease activity (>5.1), as previously described [15]. Similarly, SDAI categories were remission (≤3.3), moderate (≤11), high (≤26), and high (>26); and CDAI was categorized in remission (≤2.8), moderate (≤10), high (≤22), and high (>22) [16]. The CV risk score SCORE2 was calculated according to the 2021 European Society of Cardiology Guidelines on CV disease prevention in clinical practice [17]. SCORE2 risk categories are divided into low to moderate, high, and very high depending on different age groups (<50, 50–69, and ≥70 years). SCORE2 estimates an individual’s 10-year risk of fatal and non-fatal CV disease events in individuals aged 40 to 69 years. For healthy people aged ≥70 years, the SCORE2-OP (older persons) algorithm estimates 5-year and 10-year fatal and non-fatal CV disease events.

### 2.3. Carotid Ultrasound Assessment

Carotid ultrasound examination was used to assess carotid intima media thickness (cIMT) in the common carotid artery and to detect focal plaques in the extracranial carotid tree in patients with RA [18]. A commercially available scanner, the Esaote Mylab 70 (Genoa, Italy), equipped with a 7–12 MHz linear transducer and using an automated software-guided radiofrequency technique—Quality Intima Media Thickness in real-time (QIMT, Esaote, Maastricht, Holland)—was used for this purpose. As previously reported [18], based on the Mannheim consensus, plaque criteria in the accessible extracranial carotid tree (common carotid artery, bulb, and internal carotid artery) were defined as follows: a focal protrusion in the lumen measuring at least cIMT > 1.5 mm; a protrusion at least 50% greater than the surrounding cIMT; or arterial lumen encroaching >0.5 mm [19].

### 2.4. Statistical Analysis

Demographic and clinical characteristics in patients with RA were described as mean (standard deviation–SD) or percentages for categorical variables. For non-normally distributed continuous variables, data were expressed as the median and interquartile range (IQR). Univariable differences between groups were assessed using Student’s *t*-test, the Mann–Whitney U-test, the Chi-squared test, or Fisher’s exact test according to the normal distribution or the number of subjects. Multivariable linear regression analysis, adjusting for confounders, was assessed to analyze the association between disease-related data and blood composite scores. Confounding variables were selected from demographics and traditional CV risk factors if they had a *p*-value lower than 0.20 in the univariable relationship with RDW. All the analyses used a 5% two-sided significance level and were performed using Stata software, version 17/SE (StataCorp, College Station, TX, USA). *p*-values < 0.05 were considered statistically significant.

## 3. Results

### 3.1. Demographic and Disease-Related Data

A total of 430 patients with RA and 208 controls were included in this study. The demographic- and disease-related characteristics of the participants are shown in Table 1. Most of the participants were women (80% in both populations, *p* = 0.30), with a mean age ± SD of 56 ± 17 years in controls and 55 ± 10 in RA patients (*p* = 0.69). The mean body mass index was significantly higher in controls compared to patients. Classic CV risk factors were common in both patients and controls, and no significant differences were observed except for diabetes, which was more frequent in controls.

Regarding the results of carotid ultrasound performed in patients with RA, the mean cIMT was 0.696 ± 0.131 mm, and 42% had carotid plaques. Furthermore, the use of statins (*p* = 0.26) and aspirin (*p* = 0.061) did not differ between controls and patients (Table 1).

The median duration of the disease in this series of patients with RA was 8 (IQR 4–15) years. The mean values of CRP and ESR, at the time of the study, were 2.7 (IQR 1.3–6.1) mg/L and 18 (IQR 7–32) mm/first hour, respectively. Seventy-two percent of patients were positive for rheumatoid factor, and 65% for anti-citrullinated protein antibodies. Disease activity measured by DAS28-ESR was 3.13 ± 1.35. Thirty-six percent of the patients were being treated with prednisone and 87% were taking at least one conventional disease-modifying antirheumatic drug of any type, with methotrexate being the most widely used (73%). Nineteen percent of patients were receiving antitumor necrosis factor therapies. The frequency of use of other treatments and historical data related to the disease are shown in Table 1.

### 3.2. Multivariable Analysis of the Differences between Patients and Controls in Red Cells Count including RDW

Red blood cell count values for controls and patients are shown in Table 2. Some differences were observed in the univariable analysis. In this regard, RA patients showed significantly higher levels of mean corpuscular volume, mean corpuscular hemoglobin concentration, and RDW, but lower red blood cell counts and hematocrit than controls. However, the number of platelets and leukocytes did not differ between both groups.

A multivariate analysis was performed including all those demographic variables that differed between controls and patients with a *p*-value less than 0.20 (body mass index, smoking, hypertension, diabetes, and aspirin use). Other red blood cell count parameters were not included in the multivariable analysis due to high collinearity with RDW. After this adjustment, the RDW maintained a significantly higher value in RA patients compared to controls (beta coefficient 1.0 [95% confidence interval 0.2 to 1.8] %, *p* = 0.020) (Table 2).

### 3.3. Relationship of Demographics and Disease Related to RDW in Patients with RA

Univariable and multivariable relationships of demographic and disease-related characteristics of RA patients with RDW are shown in Table 3. In univariable analysis, age, presence of diabetes, and hypertension were significantly associated with higher levels of RDW. Regarding disease manifestations, ESR showed a positive and significant relationship with RDW after multivariable adjustment. This was not the case for other acute-phase reactants such as CRP and IL-6. The presence of rheumatoid factor and anti-citrullinated protein antibodies, and tender or swollen joints, were not related to RDW levels. Moreover, the DAS28-ESR activity index, when considered continuously, disclosed a significant and positive relationship with RDW after adjustment. Also, patients with moderate and high activity, using this score, showed higher levels of RDW compared to those who were in remission. However, no relationship was found between RDW and other activity scores such as DAS28-PCR, SDAI, and CDAI. Regarding the therapies used for the disease, only the use of tocilizumab showed a relationship, in this case negative, with RDW (Table 3).

### 3.4. Relationship of CV Risk Parameters to RDW in RA Patients

The association of subclinical carotid atherosclerosis, SCORE2 algorithm, lipid profile, and insulin resistance indices with RDW is shown in Table 4. Patients with carotid plaques had higher levels of RDW in univariable analysis. However, this relationship was not confirmed after adjusting for age, hypertension, and diabetes. The value of the CV SCORE2 risk calculator, as continuous, was positively associated with higher levels of RDW. When the relationship of SCORE2 was analyzed using this variable as ordinal, patients in the high and very high CV risk categories showed significantly higher levels of RDW compared to those included in the low or moderate risk categories (this relationship of SCORE2 with RDW was not adjusted for covariates since this index is already the result of the combination of different variables in its formula).

Regarding the lipid profile, after adjustment for covariates, ADE showed a significant and negative relationship with total cholesterol and LDL-cholesterol. On the contrary, apolipoprotein C-III and RDW were positively and significantly related. The association between RDW and insulin resistance indices was only evaluated in non-diabetic patients and if glucose was less than 110 mg/dL (n = 339). Noteworthily, RDW was independently associated with lower insulin sensitivity, higher levels of C-peptide, and an index of beta cell function (Table 4).

## 4. Discussion

The present work assessed the potential association of RDW with a wide range of clinical and laboratory characteristics in patients with RA. Our findings demonstrate that RDW is increased in the RA population compared to controls. However, its relationship with RA systemic inflammation or disease activity is uncertain. Remarkably, RDW shows a relationship with subclinical CV disease factors such as dyslipidemia and insulin resistance, and the CV risk algorithm SCORE2.

In a previous report, RDW was found to be higher in 222 patients with RA than in 126 healthy controls. Furthermore, RDW showed a positive correlation with CRP but not with ESR. Similarly, in a study of 160 RA patients, RDW was positively correlated with swollen and tender joint count, CRP, ESR, and DAS28-ESR [20]. Likewise, the level of RDW was significantly increased in 670 RA patients compared to 899 healthy donors [21], and it was positively associated with inflammatory markers such as CRP, ESR, TNF alpha, and IL-6 but negatively associated with IL-10. However, the relationship of composite disease activity scores with RDW was not evaluated in this work. Finally, in another study in 100 RA patients, RDW was positively correlated with DAS28-ESR and pain score [22]. However, it is important to note that all of these studies mentioned above had in no case performed a multivariable analysis. Furthermore, they considered disease activity using the DAS28-ESR score without taking into account other indices calculated with different acute phase reactants.

In our study, we found a positive and independent relationship between RDW and DAS28-ESR disease activity score. However, this was not the case for other disease activity scores that use CRP in their formula (DAS28-CRP and SDAI). Furthermore, the CDAI, which does not include acute phase reactants in its calculation and is based solely on tender and swollen joints, did not show an association with RDW. This is also the case for acute phase reactants, as RDW was related to ESR but not to CRP and IL-6.

ESR is commonly assessed by the Westergren method, which measures the millimeters by which red blood cells from anticoagulated whole blood drop to the bottom of a standardized, vertical, elongated tube over one hour due to the influence of gravity. Therefore, it is somehow expected to find a relationship between this red blood cell marker RDW and ESR. Thus, we believe that the relationship between RDW and DAS28-ESR is driven by the high correlation between ESR and RDW, and not by a clear effect of disease activity on RDW. This is further supported by the fact that tender and swollen joint counts and disease activity scores that do not include ESR in their formulas did not reveal a relationship with RDW.

We also found a negative relationship between the use of tocilizumab and RDW. However, the causal direction of this association cannot be correctly inferred due to the cross-sectional design of our study, and the low number of patients under this biologic drug. Additionally, leucopenia, lymphopenia, and polycythemia have been reported after tocilizumab treatment [23,24]. It is possible that the decrease in RDW has to do with this tocilizumab effect. Prospective studies are needed to analyze the exact effect of tocilizumab on RDW levels.

We also observed a negative relationship between RDW and total cholesterol and LDL-cholesterol levels. It is known that in RA there are paradoxically low levels of these lipid profile molecules [25]. The fact that a high RDW is associated with low levels of these lipid molecules is consistent with the direction that the lipid profile usually has in patients with RA. Further, apolipoprotein C-III was positively and significantly associated with RDW. It has been described that apolipoprotein C-III levels are raised in patients with RA compared to controls [26]. Therefore, both RDW markers and apolipoprotein C-III were associated with the same trend in patients compared to controls. This implies that this elevation may be related to shared disease-related mechanisms. This could also be the case for the relationship between insulin resistance and RDW. In this regard, we found a positive association of RDW with higher levels of insulin resistance and beta cell function. With respect to this, we describe for the first time this relationship with RA, reported in the general population. Consistent with our data, in a previous report, RDW was associated with beta cell function assessed by HOMA2%B after adjusting for covariates in 559 patients with type 2 diabetes [27].

Currently, there is evidence supporting the relationship between RDW and CV diseases in the general population, including stroke, peripheral arterial disease, hypertension, acute coronary syndromes, heart failure, and atrial fibrillation [28]. Considering also that RA is a condition associated with accelerated atherosclerosis and increased risk of subclinical CV disease, the positive relationship between RDW and SCORE2 found in our series of patients with RA may have potential relevance. For this reason, RDW may represent a biomarker of subclinical CV disease in patients with RA. In line with this, the increase in RDW at the time of diagnosis was able to predict the occurrence of CV events in a series of 160 patients with RA [20]. Furthermore, in this series, an increase in RDW during the first year was associated with poor CV outcomes [20].

RDW has also been studied in other immune-mediated diseases. For example, it has been linked to disease activity in patients with systemic lupus erythematosus irrespective of anemia status [29,30]. Similarly, RDW has been shown to be increased in patients with ankylosing spondylitis compared to controls and to correlate with acute phase reactants and disease activity scores in this population [31]. RDW in patients with primary Sjögren syndrome was shown to be associated with disease activity in one study [32]. However, a recent review on this topic concluded that the clinical utility of RDW in assessing disease activity in rheumatic conditions warrants more rigorous investigation through well-designed prospective studies [33].

Our work has several clinical implications. We believe that RDW would not be useful for monitoring disease activity. This is based on the fact that although it showed a relationship with ESR, this was not the case for other acute phase reactants or joint inflammation counts. Prospective studies are needed to define its usefulness in the response to treatments or how its levels progress throughout the evolution of the disease. Despite this, RDW could be a marker of high cardiovascular risk in RA given its consistent relationship with SCORE2.

We acknowledge several limitations in our study. In this regard, ESR values were not available in controls. For this reason, it was not possible to adjust the difference between patients and controls in RDW for this variable. However, it is important to highlight that at the time of the study, the ESR values in this series of RA were not particularly high (median 18 mm/first hour). Hence, we believe that the confounding effect of ESR should have been small. On the other hand, we did not assess iron or ferritin levels in our patients. For this reason, the effect that iron levels could have exerted on our findings cannot be concluded. However, at the time of recruitment, we excluded patients with aplasia or any hematological diseases from the study.

In conclusion, RDW values are higher in RA patients compared to matched controls. Although the relationship of RDW with disease activity was not consistent, RDW shows associations with traditional CV disease risk factors, including dyslipidemia and insulin resistance, and with the SCORE2 CV disease-risk prediction algorithm.

## Figures and Tables

**Table 1:** Demographics and CV risk factors in patients with RA and controls and RA disease-related data.

	Controls	Rheumatoid Arthritis	
	(n = 208)	(n = 430)	*p*
Age, years	56 ± 17	55 ± 10	0.69
Female, n (%)	**162 (79)**	**350 (81)**	**0.30**
BMI, kg/m^2^	**31 ± 3**	**29 ± 15**	**0.034**
Cardiovascular risk factors and data			
Current smoker	35 (17)	93 (22)	0.16
Obesity	60 (29)	137 (32)	0.44
Hypertension	85 (41)	148 (34)	0.11
Diabetes Mellitus	**39 (19)**	**54 (13)**	**0.031**
Dyslipidemia	164 (79)	332 (77)	0.64
Statins, n (%)	58 (28)	139 (32)	0.26
Aspirin, n (%)	16 (8)	24 (10)	0.061
Carotid ultrasound			
cIMT, mm		0.696 ± 0.131	
Carotid plaque, n (%)		180 (42)	
Disease related data			
Disease duration, years		8 (4–15)	
CRP at time of study, mg/L		2.7 (1.3–6.1)	
ESR at time of study, mm/1 h		18 (7–32)	
IL-6, pg/mL		5.0 (3.2–8.6)	
Rheumatoid factor, n (%)		303 (72)	
ACPA, n (%)		253 (65)	
Swollen joints count, n		0 (0–1)	
Tender joints count, n		1 (0–4)	
DAS28-ESR		3.13 ± 1.35	
DAS28-PCR		2.73 ± 1.08	
SDAI		12 (7–19)	
CDAI		8 (4–14)	
History of extraarticular manifestations, n (%)		38 (10)	
Erosions, n (%)		166 (43)	
Current drugs, n (%)			
Prednisone		155 (36)	
Prednisone doses, mg/day		5 (3–5)	
NSAIDs		194 (45)	
DMARDs		373 (87)	
Methotrexate		316 (73)	
Leflunomide		94 (22)	
Hydroxychloroquine		45 (18)	
Salazopyrin		28 (7)	
Anti TNF therapy		83 (19)	
Tocilizumab		23 (5)	
Rituximab		7 (2)	
Abatacept		12 (3)	
JAK inhibitors		20 (5)	

Data represent mean ± SD or median (IQR) when data were not normally distributed. CRP: C reactive protein; ACPA: Anti-citrullinated protein antibodies. NSAID: Nonsteroidal anti-inflammatory drugs; DMARD: disease-modifying antirheumatic drug. TNF: tumor necrosis factor; Obesity; ESR: erythrocyte sedimentation rate. SCORE: Systematic Coronary Risk Evaluation. JAK: Janus kinase. BMI: body mass index; DAS28: Disease Activity Score in 28 joints. CDAI: Clinical Disease Activity Index; SDAI: Simple Disease Activity Index. cIMT: carotid intima media thickness. Carotid ultrasound was not available for controls. Dyslipidemia was defined if one of the following was present: total cholesterol > 200 mg/dL, triglycerides > 150 mg/dL, HDL cholesterol < 40 in men or <50 mg/dL in women, or LDL cholesterol > 130 mg/dL. Significant *p* values are depicted in bold.

**Table 2:** Multivariable analysis of the differences between patients and controls in complete hemogram cell count.

	Controls	RA Patients			
	(n = 208)	(n = 430)	*p*	Beta Coef. (95%CI), *p*	
	Univariable			Multivariable	
Red blood cells, ×10^6^/mm^3^	**4.71 ± 0.45**	**4.51 ± 0.40**	**<0.001**	**−0.2 (−0.4–(−0.1))**	**0.001**
Hemoglobin, g/dL	13.7 ± 1.4	13.6 ± 1.3	0.099	−0.5 (−1–0−0.01)	0.055
Hematocrit, %	**42.3 ± 3.8**	**41.5 ± 3.7**	**0.011**	−1 (−3–0.06)	0.61
Mean corpuscular volume, fL	**90 ± 6**	**92 ± 6**	**<0.001**	2 (−0.08–4)	0.059
Mean corpuscular hemoglobin, pg	**29 ± 2**	**30 ± 2**	**<0.001**	**0.6 (−0.3–2)**	**0.017**
Mean corpuscular hemoglobin concentration, g/dL	32 ± 1	33 ± 2	0.68		
Red Cell Distribution Width, %	**13.5 ± 1.6**	**14.2 ± 2.2**	**<0.001**	**1.0 (0.2–1.8)**	**0.020**
Leucocytes/mm^3^	7360 ± 1879	7158 ± 2144	0.25		
Platelets, ×10^3^ mm^3^	264 ± 60	260 ± 64	0.44		

Data represent mean ± SD. In the multivariable analysis, controls are considered the reference variable. Multivariable analysis is adjusted for body mass index, hypertension, diabetes, and aspirin intake. Significant *p*-values are depicted in bold.

**Table 3:** Relationship of demographics and disease related to RDW in RA patients.

	RDW, %			
	Beta Coefficient (95%CI), *p*			
	Univariable		Multivariable	
Age, years	**0.03 (0.01–0.05)**	**0.002**		
Female	−0.2 (−0.7–0.3)	0.46		
BMI, kg/m^2^	0.0007 (−0.01–0.1)	0.92		
Cardiovascular risk factors				
Current smoker	−0.2 (−0.7–0.3)	0.39		
Obesity	0.2 (−0.2–0.7)	0.30		
Hypertension	**0.6 (−0.2–1)**	**0.008**		
Diabetes Mellitus	**0.9 (−0.3–2)**	**0.005**		
Dyslipidemia	−0.07 (−0.6–0.4)	0.77		
Statins	0.3 (−0.2–0.7)	0.20		
Aspirin	0.1 (−1–1)	0.79		
Disease related data				
Disease duration, years	0.009 (−0.01–0.03)	0.43		
CRP, mg/L	0.01 (−0.003–0.03)	0.12	0.01 (−0.004–0.03)	0.14
ESR, mm/first hour	**0.02 (0.004–0.03)**	**0.004**	**0.01 (0.003–0.02)**	**0.014**
IL-6, pg/ml	**−0.01 (−0.03–(−0.0001))**	**0.048**	−0.01 (−0.03–0.0004)	0.057
Rheumatoid factor	0.06 (−0.4–0.5)	0.79		
ACPA	−0.03 (−0.5–0.4)	0.91		
Swollen joints count, n	0.02 (−0.1–0.1)	0.81		
Tender joints count, n	−0.005 (−0.07–0.05)	0.86		
DAS28-ESR	**0.2 (0.05–0.3)**	**0.008**	**0.2 (0.03–0.3)**	**0.020**
Remission, n = 102	ref.		ref.	
Low, n = 55	0.3 (−0.2–0.8)	0.24	0.3 (−0.2–0.8)	0.25
Moderate, n = 118	**0.5 (0.1–1.0)**	**0.016**	**0.5 (0.07–0.9)**	**0.022**
High, n = 35	0.4 (−0.2–1)	0.20	0.3 (−0.4–1)	0.35
Moderate and high, n = 153	**0.5 (0.1–0.9)**	**0.013**	**0.5 (0.06–0.9)**	**0.025**
DAS28-PCR	0.1 (−0.07–0.3)	0.23		
Remission, n = 154	ref.			
Low, n = 55	0.4 (−0.1–0.9)	0.15		
Moderate, n = 88	0.4 (−0.06–0.8)	0.092		
High, n = 11	−0.03 (−1–1)	0.96		
Moderate and high, n = 99	0.3 (−0.09–0.8)	0.15		
SDAI	0.007 (−0.006–0.02)	0.30		
Remission, n = 19	ref.			
Low, n = 106	0.4 (−0.4–1)	0.31		
Moderate, n = 142	0.6 (−0.3–1)	0.17		
High, n = 40	1 (−0.01–2)	0.052		
Moderate and high, n = 182	0.7 (−0.2–1)	0.11		
CDAI	−0.0009 (−0.02–0.02)	0.94		
Remission, n = 55	ref.			
Low, n = 137	0.05 (−0.5–0.6)	0.84		
Moderate, n = 94	0.06 (−0.5–0.6)	0.84		
High, n = 24	0.07 (−0.8–0.9)	0.87		
Moderate and high, n = 118	0.06 (−0.5–0.6)	0.82		
History of extraarticular manifestations	−0.6 (−1–0.1)	0.089	−0.6 (−1–0.08)	0.084
Erosions	−0.06 (−0.5–0.4)	0.78		
Current drugs				
Prednisone	0.3 (−0.1–0.8)	0.14	0.3 (−0.1–0.7)	0.15
Prednisone doses, mg/day	−0.01 (−0.1–0.08)	0.80		
NSAIDs	0.1 (−0.3–0.5)	0.62		
DMARDs	0.2 (−0.5–0.8)	0.61		
Methotrexate	**0.5 (−0.03–1.0)**	**0.037**	0.4 (−0.02–0.9)	0.061
Leflunomide	−0.5 (−0.6–0.5)	0.83		
Hydroxychloroquine	−0.4 (−1–0.3)	0.26		
Salazopyrin	−0.5 (−1–0.4)	0.31		
Anti TNF therapy	−0.3 (−0.8–0.2)	0.25		
Tocilizumab	**−1 (−2–(−0.2))**	**0.016**	**−1 (−2–(−0.06))**	**0.036**
Rituximab	0.2 (−1–2)	0.82		
Abatacept	0.2 (−1–2)	0.76		
JAK inhibitors	−0.5 (−1–0.5)	0.35		

Data represent mean ± SD or median (IQR) when data were not normally distributed. RDW is considered the dependent variable in this analysis. RDW: Red cell distribution width, %. NSAID: Nonsteroidal anti-inflammatory drugs; DMARD: disease-modifying antirheumatic drug. TNF: tumor necrosis factor; Obesity; ESR: erythrocyte sedimentation rate, JAK: Janus kinase. BMI: body mass index; DAS28: Disease Activity Score in 28 joints. CRP: C reactive protein. ACPA: Anti-citrullinated protein antibodies; HOMA: homeostatic model assessment. CDAI: Clinical Disease Activity Index; SDAI: Simple Disease Activity Index. IL-6: interleukin 6. Dyslipidemia was defined if one of the following was present: total cholesterol > 200 mg/dL, Triglycerides > 150 mg/dL, HDL cholesterol < 40 in men or <50 mg/dL in women, or LDL cholesterol > 130 mg/dL. Multivariable analysis is adjusted for age, hypertension, and diabetes. Significant *p*-values are depicted in bold.

**Table 4:** Relationship of cardiovascular risk parameters to RDW in RA patients.

		RDW, %			
		Beta Coefficient (95%CI), *p*			
		Univariable		Multivariable	
Carotid ultrasound					
cIMT, mm	0.0696 ± 0.131	1 (−0.1–3)	0.073	0.2 (−2–2)	0.82
Carotid plaque, n (%)	180 (42)	**0.5 (0.1–1)**	**0.015**	0.3 (−0.2–0.7)	0.28
SCORE2					
SCORE2, %	3.6 (1.8–5.8)	**0.088 (0.03–0.1)**	**0.003**		
Low or moderate risk	282 (66)	ref.			
High risk	112 (26)	**0.5 (0.05–1)**	**0.032**		
Very high risk	36 (8)	0.6 (−0.1–1)	0.11		
High and very high	148 (34)	**0.6 (0.1–1)**	**0.013**		
Lipid profile					
Total cholesterol, mg/dL	206 ± 38	**−0.006 (−0.01–(−0.0002))**	**0.042**	**−0.006 (−0.01–(−0.0004))**	**0.037**
Triglycerides, mg/dL	147 ± 86	0.002 (−0.00006–0.005)	0.056	0.001 (−0.001–0.004)	0.35
HDL-cholesterol, mg/dL	56 ± 15	−0.007 (−0.02–0.007)	0.34		
LDL-cholesterol, mg/dL	120 ± 34	**−0.009 (−0.01–(−0.003))**	**0.005**	**−0.008 (−0.01–(−0.002))**	**0.011**
LDL:HDL cholesterol ratio	2.27 ± 0.93	−0.1 (−0.3–0.1)	0.33		
Non-HDL cholesterol, mg/dL	149 ± 39	−0.005 (−0.01–0.0009)	0.10	−0.005 (−0.01–0.0003)	0.064
Lipoprotein (a), mg/dL	34 (11–107)	0.0009 (−0.002–0.004)	0.53		
Apolipoprotein A1, mg/dL	174 ± 31	−0.006 (−0.01–0.0009)	0.090	−0.006 (−0.01–0.0008)	0.084
Apolipoprotein B, mg/dL	107 ± 43	−0.001 (−0.006–0.004)	0.68		
ApoB:Apo A1 ratio	0.63 ± 0.24	0.2 (−0.7–1)	0.71		
Apolipoprotein CIII, mg/dL	4.8 (2.2–8.7)	**0.1 (0.06–0.1)**	**<0.001**	**0.09 (0.05–0.1)**	**<0.001**
Atherogenic index	3.88 ± 1.28	0.02 (−0.1–0.2)	0.84		
Insulin resistance indices *					
Glucose, mg/dL	87 ± 10	**0.02 (0.001–0.05)**	**0.040**	0.02 (−0.003–0.04)	0.083
Insulin, µU/mL	7.7 (5.2–12.4)	0.02 (−0.001–0.05)	0.061	0.02 (−0.002–0.04)	0.069
C-peptide, ng/mL	2.32 (1.51–3.44)	**0.2 (0.1–0.3)**	**<0.001**	**0.2 (0.1–0.4)**	**<0.001**
HOMA2-IR	1.00 (0.66–1.56)	**0.2 (0.002–0.4)**	**0.048**	0.2 (−0.006–0.4)	0.056
HOMA2-S%	118 ± 76	**−0.003 (−0.006–(−0.0004))**	**0.026**	**−0.003 (−0.006–(−0.0003))**	**0.031**
HOMA2-B%-C-peptide	165 ± 75	**0.003 (0.0003–0.006)**	**0.031**	**0.003 (−0.0002–0.006)**	**0.036**

Data represent mean ± SD or median (IQR) when data were not normally distributed. RDW: Red cell distribution width. RDW is.the dependent variable in this analysis. * Insulin resistance analysis is only performed for non-diabetic patients and if glucose is lower than 110 mg/dL (n = 339). SCORE: Systematic Coronary Risk Evaluation, LDL: low-density lipoprotein; HDL: high-density lipoprotein. cIMT: carotid intima media thickness. HOMA: homeostatic model assessment, CI: confidence interval. LDL: low-density lipoprotein; HDL: high-density lipoprotein. Multivariable analysis is adjusted for age, hypertension, and diabetes. SCORE2 calculator relation to RDW is not adjusted for covariates. Significant *p*-values are depicted in bold.

## Data Availability

The data sets used and/or analyzed in the present study are available from the corresponding author upon request.
